# Second-Harmonic Generation Imaging Reveals Changes in Breast Tumor Collagen Induced by Neoadjuvant Chemotherapy

**DOI:** 10.3390/cancers14040857

**Published:** 2022-02-09

**Authors:** Danielle E. Desa, Wencheng Wu, Robert M. Brown, Edward B. Brown, Robert L. Hill, Bradley M. Turner, Edward B. Brown

**Affiliations:** 1 Department of Biomedical Engineering, Hajim School of Engineering and Applied Sciences, University of Rochester, Rochester, NY 14627, USA; ddesa@ur.rochester.edu (D.E.D.); robert.brown@hflcsd.org (R.M.B.); Edward.Brown@hflcsd.org (E.B.B.IV); 2 Goergen Institute for Data Science, University of Rochester, Rochester, NY 14627, USA; wencheng.wu@rochester.edu; 3 Harmonigenic Corporation, Rochester, NY 14618, USA; robert.hill@harmonigenic.com; 4 Department of Pathology and Laboratory Medicine, University of Rochester Medical Center, Rochester, NY 14642, USA; Bradley_Turner@urmc.rochester.edu

**Keywords:** neoadjuvant chemotherapy, breast cancer, tumor microenvironment, extracellular matrix, collagen, second-harmonic generation, multiphoton microscopy

## Abstract

**Simple Summary:**

Most breast cancer deaths are due to metastases. Neoadjuvant, or pre-surgical, chemotherapy is given to shrink select aggressive breast cancers but can have unpleasant side effects and induce changes in the tumor microenvironment. This pre-surgical chemotherapy also increases one signature in breast tumors that is prognostic of metastasis. We assessed the effect of neoadjuvant chemotherapy on two other prognostic signatures derived from the tumor collagen: second-harmonic generation directionality and fiber alignment. We found that directionality changes in the tumor bulk of two breast cancer subtypes but not in the tumor/stromal interface. Fiber alignment is increased in only one breast cancer subtype. The results indicate that neoadjuvant chemotherapy affects tumor extracellular collagen in a manner specific to breast tumor subtype and alters some, but not all, prognostic signatures. This may impact the clinical utility of these signatures.

**Abstract:**

Breast cancer is the most common invasive cancer in women, with most deaths attributed to metastases. Neoadjuvant chemotherapy (NACT) may be prescribed prior to surgical removal of the tumor for subsets of breast cancer patients but can have diverse undesired and off-target effects, including the increased appearance of the ‘tumor microenvironment of metastasis’, image-based multicellular signatures that are prognostic of breast tumor metastasis. To assess whether NACT can induce changes in two other image-based prognostic/predictive signatures derived from tumor collagen, we quantified second-harmonic generation (SHG) directionality and fiber alignment in formalin-fixed, paraffin-embedded sections of core needle biopsies and primary tumor excisions from 22 human epidermal growth factor receptor 2-overexpressing (HER2+) and 22 triple-negative breast cancers. In both subtypes, we found that SHG directionality (i.e., the forward-to-backward scattering ratio, or F/B) is increased by NACT in the bulk of the tumor, but not the adjacent tumor-stroma interface. Overall collagen fiber alignment is increased by NACT in triple-negative but not HER2+ breast tumors. These results suggest that NACT impacts the collagenous extracellular matrix in a complex and subtype-specific manner, with some prognostic features being unchanged while others are altered in a manner suggestive of a more metastatic phenotype.

## 1. Introduction

Breast cancer is the most common malignancy in women, with 1 in 8 being diagnosed at some point during their lifetime [1]. Neoadjuvant chemotherapy (NACT) is an option for subsets of breast cancer patients to shrink large and aggressive tumors prior to surgical removal. NACT is typically administered for 3–6 months, and achieving pathologic complete response (pCR) to NACT is associated with better long-term outcomes, particularly for human epidermal growth factor 2-overexpressing (HER2+) and triple-negative breast cancers (TNBC) [2,3,4,5,6,7].

While clinical trials have shown that NACT can successfully downsize breast tumors, NACT also has certain disadvantages. NACT is associated with a risk for local recurrence [8] and is also known to induce changes within the tumor microenvironment, such as increased tumor angiogenesis, prolonged inflammation, and cellular stress [9,10,11,12]. One example of NACT’s undesirable effects involves the multicellular complex called a “tumor microenvironment of metastasis” (TMEM) [13]. TMEMs are observed on immunolabeled tumor sections and comprise three cell types: an endothelial cell, a perivascular macrophage, and a tumor cell expressing Mammalian-enabled (MENA), an actin protein that regulates cell adhesion and motility [14,15]. It is thought that vascular permeability is transiently regulated by macrophages, allowing tumor cell dissemination at TMEM structures [14]. TMEM density is prognostic of distant organ metastasis in human breast cancers [15] and NACT has been shown to increase TMEM formation [16,17]. This observation has led us to ask if NACT alters other image-based prognostic signatures in the breast tumor microenvironment.

One constituent of the tumor microenvironment is the extracellular matrix (ECM), which is characterized by increased stromal protein levels, stiffness, and changes in the overall organization relative to the healthy breast. These changes cause aberrant signaling, create dense physical barriers, and increase interstitial pressure, directly impacting chemotherapy transport and hence efficacy [18,19]. This is evidenced by the fact that the percentage of the intratumoral stroma is prognostic of disease-free survival in a cohort of invasive breast cancers [20]. Fibrillar collagen, a major component of the tumor ECM, can produce an intrinsic optical signal called a second-harmonic generation (SHG). This nonlinear optical process occurs when two photons scatter off a noncentrosymmetric material (such as a collagen fiber), producing a single photon with exactly twice the energy of the initial photons. SHG imaging of tumor collagen fibers readily reveals their orientation, which provides useful information prognostic of metastasis and predictive of NACT efficacy: the tendency of fibers to align orthogonally to the tumor border (known as “TACS-3”) is prognostic of disease-free survival in breast cancer patients [21,22], as is the uniformity of those fibers in SHG collagen images [23]. Furthermore, overall collagen fiber organization (i.e., alignment in a common direction) is prognostic of lymphatic metastasis and predictive of response to NACT [6], as well as prognostic of overall disease-free survival [24].

In addition to providing information about individual collagen fiber orientation and overall organization through imaging, the directionality of SHG emission from collagen fibers is sensitive to the fiber internal structure (FIS), which is the diameter, spacing, and packing disorder of the collagen fibrils that assemble into an individual fiber [25,26,27,28]. One measure of SHG directionality is the ratio of forward-detected to backward-detected SHG photons (where “forward” is in the direction of the excitation laser), or “F/B”. F/B imaging and analysis have been applied to several clinical phenomena, including distinguishing normal ovarian tissue tumor [29,30], invasive ductal carcinoma from the healthy breast tissue and ductal carcinoma in situ [31], and interstitial fibrosis from healthy lung [32]. We have previously shown that F/B measured in the tumor-stromal interface of primary breast tumors is an independent prognostic indicator of 10-year metastasis-free survival [33,34]. Consequently, we see that collagen fiber organization and SHG directionality are both prognostic of breast cancer metastasis, as is the density of TMEMs. If NACT alters TMEMs, the question naturally arises: does NACT alter collagen fiber organization and collagen SHG directionality?

In this study, we used SHG F/B imaging to study changes induced by NACT in collagen FIS as well as collagen fiber organization. We acquired samples from two breast cancer cohorts that are typical candidates for NACT: HER2+ and TNBCs. We generated SHG F and B images in two regions: the highly cellular tumor bulk and the surrounding collagenous tumor-stroma interface. We then compared F/B as well as collagen fiber organization from these regions before and after NACT administration by imaging the pre-NACT diagnostic core needle biopsies and the paired post-NACT, post-mastectomy tumor excisions. 

## 2. Materials and Methods

### 2.1. Patient Samples

22 HER2+ and 22 TNBC patients were identified from the pathology files at the University of Rochester Medical Center (URMC) (2009–2020). See Table S1 for detailed information on patients’ age, nuclear grade, and receptor status. The studies described in this work were performed using samples from patients diagnosed and administered NACT within the last ten years; therefore, their ultimate metastatic outcome is unknown. The use of patient samples was approved by the Institutional Review Board at the University of Rochester (IRB RSRB00069270). All HER2+ patients received trastuzumab and/or pertuzumab, with a standard combination neoadjuvant ACT regimen (anthracycline, taxane, and cyclophosphamide) and/or platinum therapy. All TNBC patients received a standard ACT regimen, etoposide, 5-fluorouracil, methotrexate, and/or platinum therapy. Core needle biopsies were collected prior to NACT administration and post-NACT primary tumors were collected after mastectomy (partial or total). Both core needle biopsy samples and excision samples were treated the same, with the same buffer, same fixative, same time in fixative, etc. Naturally, the thin cylinders of tissue produced by the core needle biopsy will likely allow fixative, more ready diffusive penetrance to the middle of the tissue sample than the larger portions of tissue produced by primary tumor excisions. All tissues were processed in the URMC pathology laboratory and mounted on slides as 5-μm-thick, H&E-stained FFPE sections. H&E slides and immunohistochemistry stains were reviewed by at least two board-certified breast pathologists with the manual interpretation of HER2 (rabbit antihuman HER2, Dako HercepTest™). Fluorescence in-situ hybridization (FISH) was performed on all equivocal HER2 immunohistochemistry results (HER2 IQFISH pharmDx, FDA kit, Dako), and the FISH results were used in lieu of the immunohistochemistry for these cases.

The evaluation of the extent of residual disease following NACT was performed on the post-treatment excisions following the method of Symmans et al. [35,36]. Briefly, the gross description along with clinical imaging studies and specimen photographs were used to determine the largest dimensions of the residual primary tumor bed. Evaluation of microscopic sections was used to determine (1) the proportion of primary tumor beds and (2) the number of axillary lymph nodes that contained metastatic carcinoma as well as (3) the diameter of the largest metastatic deposit. This information was entered into the online calculator [37], and the RCB class was determined where 0 is equivalent to pCR and classes I, II, and III represent minimal, moderate, and extensive residual disease, respectively.

To facilitate placing our imaged fields in desired regions of the tumor tissue (see below), we performed these studies in formalin-fixed, paraffin-embedded (FFPE), hematoxylin, and eosin (H&E)-stained sections from needle biopsies and tumor excisions. We note that F/B values reported here are not necessarily equal to F/B that would be measured in unprocessed fresh tissues because various steps in processing and mounting may affect that F/B value. Specifically, SHG F/B is expected to decrease with FFPE processing [25,38] and H&E staining [39]. 

### 2.2. Imaging

A Spectra-Physics MaiTai Ti:Sapphire laser (circularly polarized at the sample using a Berek compensator to ensure equal excitation of all fiber orientations, 100 fs pulses at 80 MHz, 810 nm, ~8–10 mW at the sample) was directed through an Olympus Fluoview FV300 scanner. The laser was focused through an Olympus UMPLFL20XW water-immersion lens (20×, 0.95 NA), which subsequently captured backward-propagating SHG signal (i.e., the B image). This backward-propagating SHG signal was separated from the excitation beam using a 670 nm dichroic mirror, filtered (HQ405/30 m-2P, Chroma), and collected by a photomultiplier tube (Hamamatsu H10492–003). The forward-scattered SHG (i.e., the F image) was collected through an Olympus 0.9 NA condenser, reflected by a 565 nm dichroic mirror (565 DCSX, Chroma) to remove excitation light, and captured using an identical filter (HQ405/30m-2P, Chroma) and identical photomultiplier tube (Hamamatsu H10492–003). All images were 512 × 512 pixels and collected as *z*-stacks in 3 µm steps (3 slices, 6 µm total). Next, stacks were maximum intensity-projected to form a single forwards-detected image and single backward-detected image, which serves as a convenient pixel-by-pixel autofocus if the tissue is not perfectly parallel to the image plane, prior to performing the image analysis described below.

**Figure 1:**
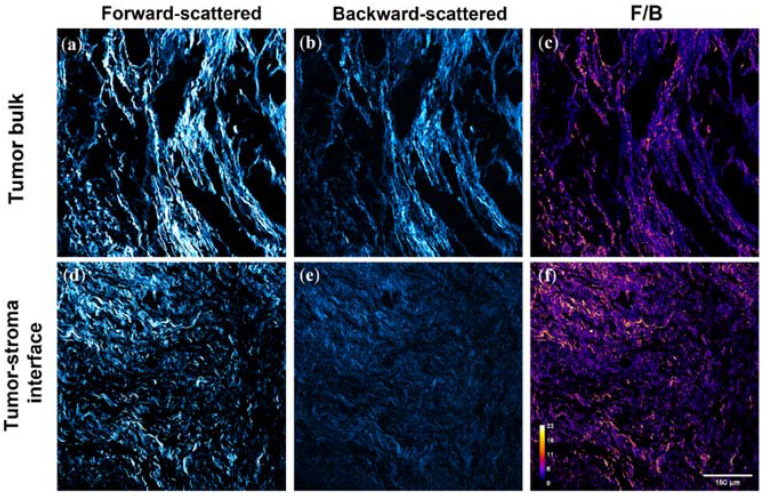

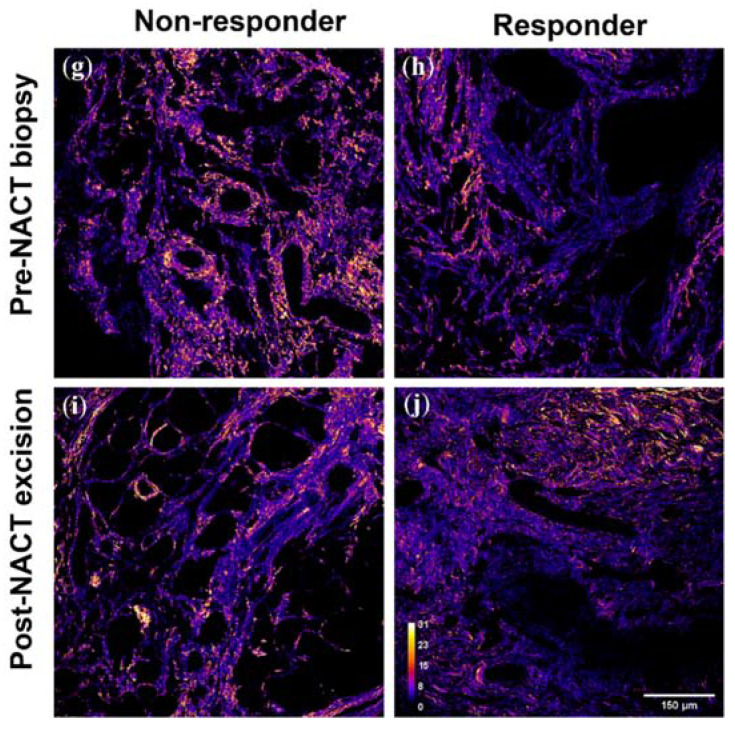
SHG imaging was performed in the tumor bulk (**a**–**c**) and adjacent tumor-stroma interface (**d**–**f**), regions containing distinctly different fibrillar collagen features. Representative forward-scattered (**a**,**d**), backward-scattered (**b**,**e**), and F/B (**c**,**f**) SHG images of one patient’s pre-NACT biopsy are shown. Imaging was performed in these regions before (**g**,**h**) and after (**i**,**j**) NACT administration, with a variety of patient responses exhibited. In patients that responded favorably to NACT, post-treatment imaging was performed in the residual tumor bed (**j**) equivalent to the tumor bulk (**i**). Collagen FIS varies in both region types, where yellow pixels represent a higher F/B value and purple represents lower F/B.

Three image pairs were first taken in the cellular “tumor bulk” of a pre-NACT core needle biopsy, followed by three image pairs taken in the collagenous “tumor-stroma interface” directly adjacent to the tumor bulk. Next, three images were taken in the tumor bulk of the matched post-NACT, post-mastectomy tumor excision. We note that several patients did respond favorably to NACT (achieving pCR), resulting in a lack of tumor cells in those excisions. In these cases, a board-certified breast pathologist marked an area containing the residual tumor bed in which the equivalent tumor bulk images were taken. We then took three images in the tumor-stroma interface adjacent to residual tumor bulk (or residual tumor bed) in these matching post-NACT excisions. Example F/B images of these regions can be seen in Figure 1.

### 2.3. Image Analysis: F/B with User-Defined Thresholds

Image pairs were analyzed using Fiji, as we have previously described [33,40]. For a given region of interest (ROI), masks for the forward (F)- and backward (B)-scattered images were created by a blinded observer selecting a threshold for each F and each B image that best distinguished pixels within fibers from background pixels. Pixels above the threshold were set to 1 and those below to 0, producing binary F and B masks. The binary masks were multiplied together to create a final mask of pixels within collagen fibers. The background-subtracted F and B images were divided to produce a single F/B image, which was multiplied by this final mask. The average value of the nonzero pixels from the resultant image yielded the average F/B of the entire ROI.

### 2.4. Image Analysis: F/B with Adaptive Thresholds

In the thresholding method described above, a user assigns a single F and B threshold value to the entire image to select pixels within collagen fibers. This was the method used in the original demonstration of F/B as an independent prognostic indicator of metastasis-free survival time [33]. To better account for spatial variation in intensity within the SHG images and to reduce the impact of possible user bias, we also employed an automated adaptive thresholding method, as previously described [33,41]. An ROI was first binarized based upon whether each pixel is greater than or less than 0.6x the average pixel intensity of the entire image. A series of progressively smaller windows were centered on each pixel in the ROI, and the percentage of nonzero pixels was calculated for each of these possible window positions. The smallest window size that produced >5% nonzero pixels when centered on any pixel in the ROI was selected for that image. This window size was applied to the original image to generate a binary mask in which all pixels were assigned a value of 1 or 0 depending upon their value relative to 0.6× the average of all pixels in that window. This algorithm was implemented in MATLAB (Mathworks, Inc., Natick, MA, USA) to produce a binary mask for each image which was then multiplied by the background-subtracted F/B image to produce a single F/B value.

### 2.5. F/B Calibration

The same disc of an H&E-stained tissue microarray was imaged hourly using the same parameters described above. All calibration images from Hour 2 and later were *x-y* registered to the Hour 1 image using MATLAB (Mathworks, Inc.). F/B was determined with a user-defined threshold and F/B from Hour 1 divided by F/B from each subsequent hour to generate a calibration factor that quantifies small variations in detector alignment or other factors that may drift over the course of an imaging session. All F/B values were then normalized by the calibration factor appropriate for their imaging session (average factor = 0.987).

### 2.6. Image Analysis: Collagen Fiber Organization

The overall variability in collagen fiber orientation (i.e., fiber organization) was quantified using a variation of the method of Dekker et al. [6]. Using two F images from the tumor bulk of each patient, a uniform 3 × 3 grid was superimposed on each F image, producing 9 intersection points. The collagen fiber closest to each intersection point was selected, and its orientation relative to the horizontal “3 o’clock” direction was measured (this variation of the method, i.e., using a 3 × 3 grid to dictate measurement locations, reduces possible biases that may be introduced if the user were allowed to freely choose numbers and locations of measurements within the image). The standard deviation of the 9 resultant fiber orientations was then calculated and used as a measure of the relative organization of the collagen in that image, with a lower standard deviation indicating more organized fiber ensembles.

### 2.7. Statistical Analysis

Statistical analysis was performed using GraphPad Prism 5. Data were first assessed for normality using the D’Agostino and Pearson omnibus normality test followed by paired or unpaired *t*-tests or their nonparametric equivalents (Wilcoxon signed-rank or Mann–Whitney) where appropriate.

## 3. Results

### 3.1. SHG F/B from the Tumor Bulk Differs from the Tumor-Stroma Interface in HER2+ Tumors

**Figure 2:**
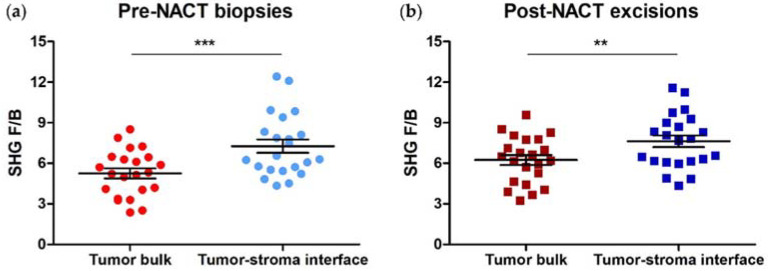
Collagen fiber internal structure differs between tumor regions in both (**a**) pre-NACT HER2+ needle biopsies and (**b**) post-NACT HER2+ tumor excisions. Each data point is an average of 3 ROI from one patient. Paired *t*-test, *** *p* = 0.0003 and ** *p* = 0.0051, respectively. Error bars = SEM, *n* = 22.

In an earlier study using excised invasive ductal carcinoma samples, we observed differences in collagen FIS, as reported by F/B, between the tumor bulk and the tumor-stroma interface [33]. In this study, we took pre-NACT core needle biopsies and matching post-NACT excisions from 22 HER2+ patients, and generated F/B values for each of 3 ROIs within each of the two types of tumor region. These were averaged to produce a single F/B for tumor bulk and one for tumor-stroma interface in each pre-NACT biopsy section and each post-NACT excision. In agreement with our previous results, we found that F/B from tumor-stroma interface (7.26 ± 0.49) is significantly higher than F/B from tumor bulk (5.25 ± 0.36) in HER2+ core needle biopsies (paired *t*-test, *p* = 0.0003, Figure 2a). This regional difference remained after NACT administration (F/B = 7.62 ± 0.43 (tumor-stroma interface) versus 6.24 ± 0.37 (tumor bulk); paired *t*-test, *p* = 0.0051, Figure 2b). Also, initial F/B in either region is not associated with RCB class when RCB class is binarized according to similar long-term patient outcome (0/I versus II/III) (logistic regression, *p* = 0.75 (tumor bulk) and *p* = 0.37 (tumor-stroma interface) [33].

### 3.2. SHG F/B increases with NACT in the Tumor Bulk but Not the Tumor-Stroma Interface of HER2+ Tumors

**Figure 3:**
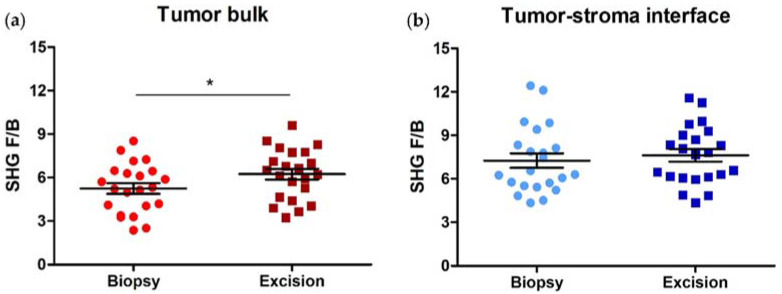
Collagen fiber internal structure is affected by NACT administration in the tumor bulk of HER2+ breast tumors. (**a**) F/B from tumor bulk significantly increases after NACT administration, while (**b**) F/B from tumor-stroma interface does not. Each data point is an average of 3 ROI from one patient. Paired *t*-test, * *p* = 0.015 and *p* = 0.54, respectively. Error bars = SEM, *n* = 22.

We next compared average F/B values from each region type between the pre-NACT biopsies and post-NACT HER2+ excisions. In cases where patients exhibited a complete response to NACT (i.e., no remaining visible tumor cells), F/B from the pre-NACT tumor bulk was compared to F/B from the post-NACT residual tumor bed. F/B significantly increases in the tumor bulk with NACT administration (5.25 ± 0.36 (biopsy) versus 6.24 ± 0.37 (excision); paired *t*-test, *p* = 0.015, Figure 3a), but not in the tumor-stroma interface of HER2+ samples (7.26 ± 0.49 (biopsy) versus 7.62 ± 0.43 (excision); paired *t*-test, *p* = 0.54, Figure 3b). 

**Figure 4:**
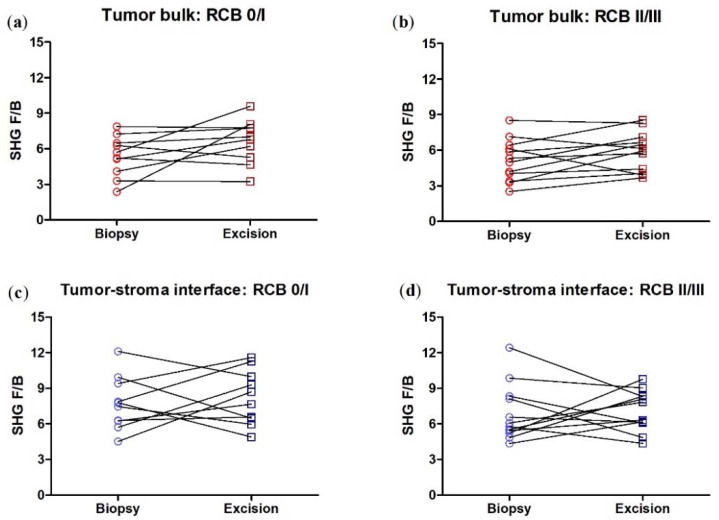
When the HER2+ patients are subdivided into RCB 0/I and RCB II/III subgroups, NACT does not produce a significant difference in F/B. Each data point is an average of 3 ROI from one patient. Paired *t*-test, tumor bulk: (**a**) *p* = 0.09, *n* = 10 and (**b**) *p* = 0.09, *n* = 12. Tumor-stroma interface: (**c**) *p* = 0.59, *n* = 10 and (**d**) *p* = 0.77, *n* = 12. Error bars = SEM.

We next broke down the patients by RCB class, pooling RCB class 0 and I together, and RCB class II and III together as they have similar five-year prognoses [35]. We found that in the tumor bulk, the change in F/B is now not quite significant in either RCB group, presumably due to the lower n produced by subdividing the patients, and in the tumor/stromal interface, any change in F/B remains insignificant in either RCB group (Figure 4).

### 3.3. SHG F/B from Tumor Bulk Differs from Tumor-Stroma Interface in TNBCs

**Figure 5:**
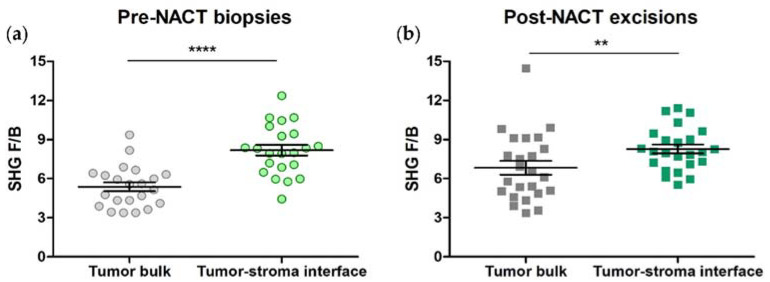
Collagen fiber internal structure, as reported by F/B, differ between tumor regions in both (**a**) pre-NACT TNBC needle biopsies and (**b**) post-NACT TNBC tumor excisions. Each data point is an average of 3 ROI from one patient. Paired *t*-test, **** *p* < 0.0001 and ** *p* = 0.0032, respectively. Error bars = SEM, *n* = 22.

We repeated the above experiments using samples from 22 TNBC patients. F/B values were generated for each of the 3 ROIs within each region type, then averaged to produce a single F/B for tumor bulk and one for tumor-stroma interface in each pre-NACT biopsy and post-NACT excision section. In agreement with previous results, we found that F/B from tumor-stroma interface (8.18 ± 0.41) is significantly higher than F/B from tumor bulk (5.37 ± 0.34) in TNBC core needle biopsies (paired *t*-test, *p* < 0.0001, Figure 5a). This regional difference remained after NACT administration (F/B = 8.11 ± 0.35 (tumor-stroma interface) versus 6.81 ± 0.56 (tumor bulk); paired *t*-test, *p* = 0.0032, Figure 5b). Initial F/B in either region is not associated with RCB class, when RCB class is binarized according to similar long-term patient outcome (0/I versus II/III) (logistic regression, *p* = 0.81 (tumor bulk), *p* = 0.78 (tumor-stroma interface) [33]. We also note that there is no difference between F/B from the tumor bulk or tumor-stroma interface of TNBCs relative to HER2+ tumors (*p* = 0.81 and 0.16, respectively.

### 3.4. SHG F/B Increases with NACT in the Tumor Bulk but Not the Tumor-Stroma Interface of TNBCs

**Figure 6:**
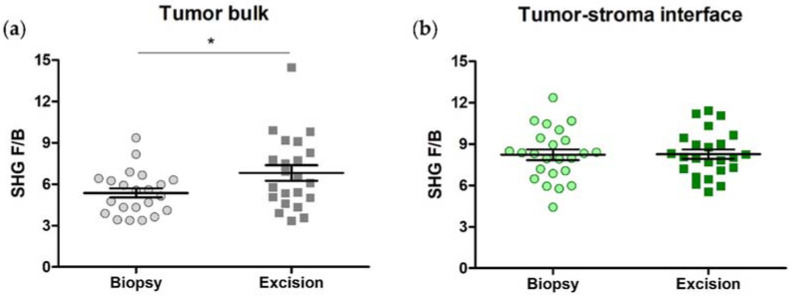
Collagen fiber internal structure, as reported by F/B, is affected by NACT administration in the tumor bulk of triple-negative breast tumors. (**a**) F/B from tumor bulk increases after NACT administration while (**b**) F/B from tumor-stroma interface remains unchanged. Each data point is an average of 3 ROI from one patient. Paired *t*-test, * *p* = 0.038 and *p* = 0.92, respectively. Error bars = SEM, *n* = 22.

We next compared average F/B values from each region type between the pre-NACT biopsies and post-NACT excisions. Again, in those patients that exhibited a complete response to NACT, F/B from the pre-NACT tumor bulk was compared to F/B from the post-NACT tumor bed. As in the HER2+ patients, F/B significantly increases in the tumor bulk with NACT administration (5.37 ± 0.34 (biopsy) versus 6.81 ± 0.56 (excision); paired *t*-test, *p* = 0.038, Figure 6a), but not in the tumor-stroma interface of TNBC samples (8.18 ± 0.41 (biopsy) versus 8.11 ± 0.35 (excision); paired *t*-test, *p* = 0.92, Figure 6b).

**Figure 7:**
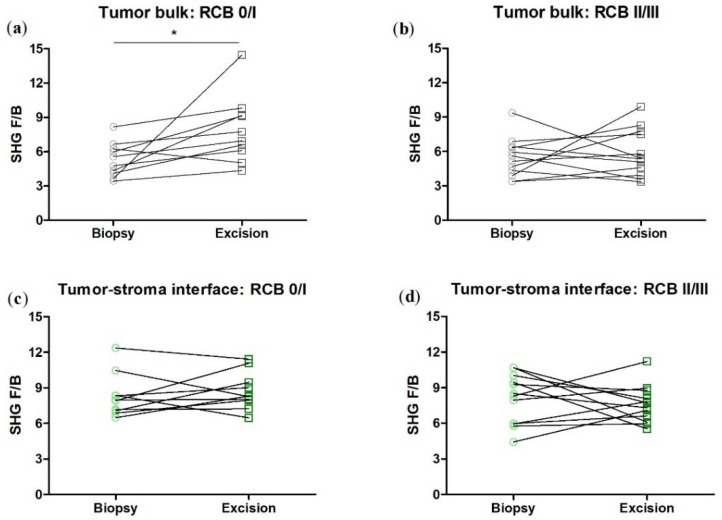
When the TNBC patients are subdivided into RCB 0/I and RCB II/III sub-groups, NACT produces a significant difference in F/B in the RCB 0/I but not RCB II/III subgroup in tumor bulk. NACT does not produce a significant difference in F/B in either subgroup in the tumor/host interface. Each data point is an average of 3 ROI from one patient. Paired *t*-test, tumor bulk: (**a**) * *p* = 0.031, *n* = 10 and (**b**) *p* = 0.57, *n* = 12. Tumor-stroma interface: (**c**) *p* = 0.47, *n* = 10 and (**d**) *p* = 0.51, *n* = 12. Error bars = SEM.

We next broke down the patients by RCB class, pooling RCB class 0 and I together, and RCB class II and III together. We found that in the tumor bulk, the change in F/B with NACT is significant in the RCB 0/I group, while there is no significant change in F/B in the RCB II/III group, nor in either RCB group in the tumor/stroma interface. (Figure 7).

### 3.5. SHG F/B Generated Using Adaptive Thresholding 

The thresholding method used above requires that a blinded observer assign a single F and B threshold to the entire image to distinguish collagen pixels from background pixels, as used in the original demonstration of F/B’s prognostic ability. To better account for heterogeneity in intensity within SHG images and minimize the risk of bias due to user selection of thresholds, we repeated the F/B analysis described above using an adaptive thresholding method. In each case, the results were the same: F/B from tumor-stroma interface is significantly higher than F/B from tumor bulk in both HER2+ (F/B = 8.95 ± 0.63 versus 6.51 ± 0.55; paired *t*-test, *p* = 0.0004 and TNBC core needle biopsies. (F/B = 9.92 ± 0.56 versus 6.73 ± 0.45; paired *t*-test, *p* < 0.0001. This regional difference remained unchanged after NACT administration in both HER2+ (F/B = 9.44 ± 0.48 (interface) versus 8.02 ± 0.56 (bulk); paired *t*-test, *p* = 0.029 and TNBC tumor excisions (F/B = 9.77 ± 0.41 (interface) versus 8.37 ± 0.62 (bulk); paired *t*-test, *p* = 0.0073.

We then compared average F/B values generated using adaptive thresholding from each region type between the pre-NACT biopsies and post-NACT excisions. In HER2+ samples, we again found that F/B from tumor bulk significantly increased in excisions (8.02 ± 0.56) relative to the matched biopsy samples (6.51 ± 0.55; paired *t*-test, *p* = 0.018). There was no significant difference between F/B from the tumor-stroma interface of pre-NACT biopsies and matched post-NACT excisions (8.95 ± 0.63 (biopsy) versus 9.44 ± 0.48 (excision); paired *t*-test, *p* = 0.51.

These results were also true of TNBC samples: F/B from tumor bulk significantly increased in excisions (8.37 ± 0.62) relative to the matched biopsy samples (6.73 ± 0.45; paired *t*-test, *p* = 0.031. There was no significant difference between F/B from the tumor-stroma interface of pre-NACT biopsies and matched post-NACT excisions (9.92 ± 0.56 (biopsy) versus 9.77 ± 0.41 (excision); paired *t*-test, *p* = 0.87).

### 3.6. Collagen Fiber Organization Changes with NACT Administration in TNBC, but Not HER2+ Tumors

**Figure 8:**
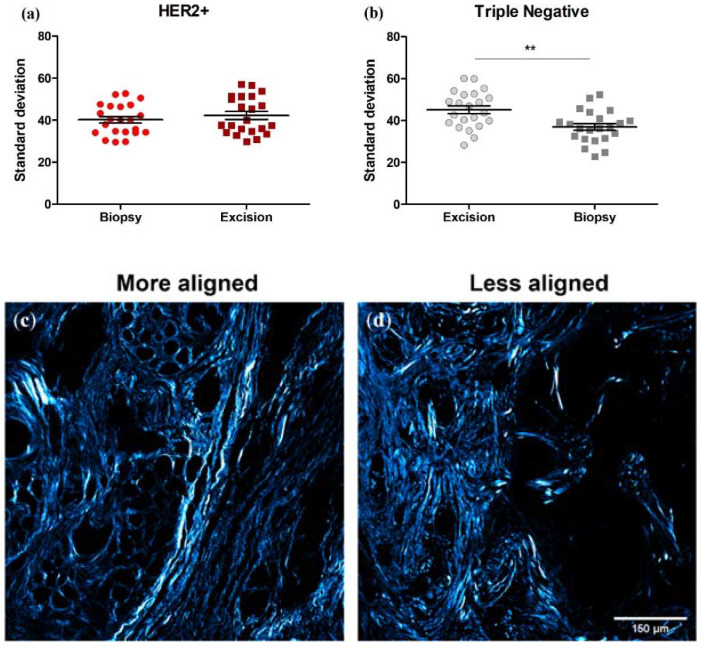
Collagen fiber organization can change with NACT administration in TNBCs. (**a**) Fiber organization is unaffected by NACT in HER2+ tumors (i.e., the standard deviation is unchanged). Paired t-test, *p* = 0.41. (**b**) In TNBCs, fiber organization increases in post-treatment excisions (i.e., the standard deviation decreases), indicating more aligned fibers after NACT. Paired t-test, ** *p* = 0.0051. Error bars = SEM, *n* = 22. (**c**,**d**) Representative images of more and less aligned collagen.

We assessed pre-NACT core needle biopsies and post-NACT tumor excisions for the relative organization of collagen fiber orientation as in Dekker et al. [6]. Initial fiber organization is not associated with RCB class (*p* = 0.77 (HER2+) and *p* = 0.43 (TNBC)) [35]. However, note that this study was performed on different tumor subtypes and with a different readout of NACT efficacy than in Dekker et al. [6]. There was no significant difference between HER2+ and TNBC fiber organization (as quantified by the standard deviation of fiber directions) either pre- or post-NACT (40.2 ± 7.32° versus 45.2 ± 8.78°, unpaired *t*-test, *p* = 0.16; and 42.3 ± 8.82° versus 36.9 ± 7.73°, unpaired *t*-test, *p* = 0.16). In HER2+ tumors, the extent of fiber organization was unchanged between matched pre- and post-NACT samples (40.2 ± 7.32° versus 42.3 ± 8.82°, paired *t*-test, *p* = 0.41, Figure 8a). However, the extent of fiber organization increased between pre- to post-NACT TNBC samples (i.e., the standard deviation decreased: 45.2 ± 8.78° versus 36.9 ± 7.73°, paired *t*-test, *p* = 0.0051, Figure 8b). In other words, fibers become more aligned in TNBCs after NACT, as seen in Figure 8c.

**Figure 9:**
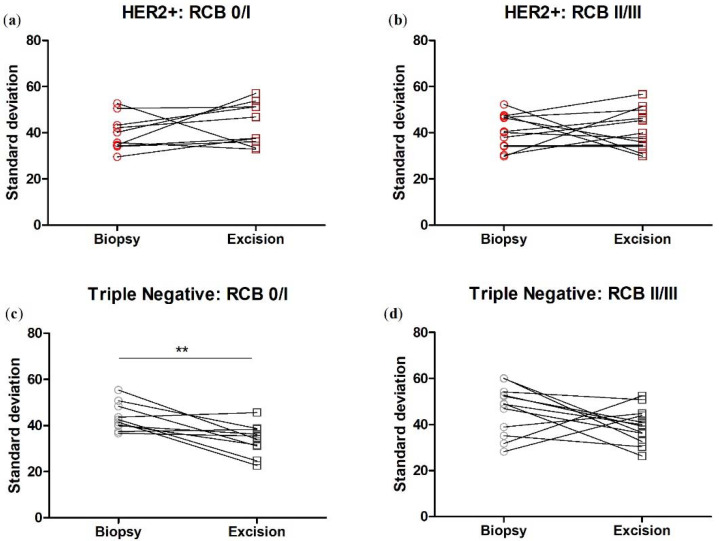
When patients are subdivided into RCB 0/I and RCB II/III sub-groups, NACT produces a significant difference in fiber angle variability in the RCB 0/I but not RCB II/III subgroup in TNBC tumors. NACT does not produce a significant difference in fiber angle variability in either subgroup in HER2+ tumors. Each data point is an average of 3 ROI from one patient. Paired *t*-test, HER2+: (**a**) *p* = 0.27, *n* = 10 and (**b**) *p* = 0.91, *n* = 12. TNBC: (**c**) ** *p* = 0.0068, *n* = 10 and (**d**) *p* = 0.14, *n* = 12. Error bars = SEM.

We next broke down the patients by RCB class, pooling RCB class 0 and I together, and RCB class II and III together. We found that in the TNBC patient samples, the change in fiber angle variability with NACT is significant in the RCB 0/I group, while there is no significant change in the RCB II/III group. There is no change in fiber angle variability in either RCB group within the HER2+ cohort (Figure 9).

## 4. Discussion

As personalized breast cancer diagnoses and treatments are continually developed and improved, it is critical to understand the potential risks and off-target effects they present. NACT is a widely used preoperative treatment regimen for patients with locally advanced and aggressive breast cancers, particularly HER2+ and TNBCs. However, not enough is known about its long-term effects on the microenvironment and subsequent impact on patient survival. For example, NACT has been shown to induce changes in the tumor microenvironment, including one known image-based prognostic marker (TMEMs), and under certain conditions may increase the risk of tumor progression [10,11,16]. In this paper, we investigated the effect of NACT on two other image-based prognostic/predictive ECM properties: collagen FIS and the relative organization of fiber orientation.

We found that NACT increased SHG scattering directionality, or “F/B,” in the tumor bulk, but not tumor-stroma interface, of both HER2+ and TNBC samples. The effect of NACT on F/B in tumor bulk was more pronounced in those tumors which demonstrated a greater response to chemotherapy: in TNBC tumors, there was a pronounced difference between the effect of NACT in RCB 0/I (*p* = 0.031) versus RCB II/III (*p* = 0.57) patients. In HER2+ tumors, this difference in effect was reduced (*p* = 0.09 in both RCB groups), perhaps due to the relatively low N produced when subdividing an already small population. Our previous work showed that F/B in the tumor-stroma interface, but not tumor bulk, is prognostic of metastasis [33]. Therefore, the fact that F/B in the tumor-stroma interface is unchanged with NACT treatment suggests that unlike in the case of TMEMs [16], NACT is not altering this prognostic signature (i.e., F/B) in a manner that produces a significantly more metastatic phenotype in either subtype.

Other studies have demonstrated that increased fiber organization (i.e., the lower standard deviation of fiber directions) is correlated with increased lymph node metastases [6]. We found that collagen organization increases in our TNBC (but not HER2+) cohort after NACT. Again, the effect was more pronounced in those tumors that demonstrated a greater response to chemotherapy: in TNBC RCB 0/I tumors *p* = 0.0068 while RCB II/III tumors *p* = 0.14. This finding suggests that, like in the case of TMEMs, NACT is altering this signature (i.e., fiber organization) to induce a more metastatic phenotype in our TNBC cohort. This is not true of the HER2+ tumors. What, if anything, these changes ultimately mean for the overall metastatic outcome and patient survival is difficult to predict, as breast cancer metastasis is a complex process, with TMEMs, collagen FIS, and collagen organization likely all playing different roles in different tumor subtypes.

The observed changes in tumor bulk F/B and collagen fiber organization could have several possible mechanisms. F/B is a measurement that is impacted by collagen fibril diameter, spacing, and disorder within the larger fiber [27,42]; therefore, an increased F/B ratio implies that one or more of these physical properties has been altered. This may occur due to tumor cells sensing and manipulating the local ECM within the tumor bulk: we have previously found that breast tumor cells alter F/B in pure collagen gels [43]. There is also evidence that tumor spheroids manipulate collagen fiber alignment via compressive remodeling [44,45]. Therefore, NACT may alter collagen FIS and organization by altering how tumor cells manipulate collagen.

NACT may also impact stromal cell behavior, causing direct or indirect collagen FIS and organization changes. For example, cancer-associated fibroblasts (CAF) are stromal cells activated by tumor-secreted factors that play a role in ECM remodeling and tumor cell survival [46]. NACT is known to stimulate CAF recruitment, leading to increased tumor ECM density and stiffness through excess stromal protein and lysyl oxidase (LOX) production [47,48]. Blocking LOX-like 2, a member of the LOX enzyme family, alters collagen fibril thickness and organization, in turn disrupting human breast tumor cell motility, adhesion, and invasion [49]. LOXL2 also directly activates CAFs in a murine breast tumor model and is upregulated in cells seeded on dense, aligned collagen matrices [46]. Taken together, these observations suggest that NACT may lead to altered collagen FIS and organization by inducing changes in tumor and stromal cells within the bulk of affected tumors.

The specific distribution of changes in collage properties across the tumor types is revealing, given the fact that the two tumor types received two different regimens of chemotherapy and that the observed changes in collagen properties tended to be found in those tumors with the greatest observed response to therapy. F/B in the tumor bulk was changed by NACT in both HER2+ and TNBC tumors. Both tumor types received an ACT regimen (anthracycline, taxane, and cyclophosphamide) and/or platinum therapy. Some of the elements of this common therapy regime are known to induce collagen changes: Cyclophosphamide and anthracycline treatment alter collagen deposition [50], as does taxane treatment [51]. Conversely, fiber angle variability is altered in TNBC tumors but not in HER2+ tumors. Both tumor types received cisplatin and a taxane, which increases the extent of collagen fiber alignment [51], consistent with our observation in TNBC tumors. The lack of change in HER2+ tumors could be explained by the fact that they alone received trastuzumab, which is known to decrease collagen fiber alignment [52]. Hence any tendency of cisplatin/taxane therapy to enhance collagen alignment may have been “canceled out” by the alignment-disrupting effects of trastuzumab.

On a cellular level, the machinery by which these collagen alterations are induced by NACT may be found in the epithelial-to-mesenchymal transition as tumor collagen properties are known to drive EMT [53,54]. On a molecular level, tumor cell invasion and drug resistance are thought to be mediated in part by integrins, key molecules in cell adhesion processes [55]. Specifically, β1 integrin is both a prognostic and predictive marker in TNBCs [56], while β1 activation and subsequent collagen upregulation are characteristic of drug-resistant HER2+ tumors [55] and mediate chemotherapy-induced apoptosis [57]. TGF- β is an inducer of the epithelial-mesenchymal transition and stimulates collagen production, potentially increasing the quantity and organization of ECM within the tumor bulk. Elevated levels of TGF-β1 are associated with lymph node metastasis [58] and promote targeted migration through the lymphatic system in animal breast tumor models [59,60], while TGF-β blockade inhibits mammary tumor growth and metastasis and improves chemotherapy efficacy [61]. This work suggests future studies in which the unique effects of each chemotherapy drug on collagen F/B and fiber ordering are further elucidated, with an eye towards the machinery of EMT as possible molecular mechanisms.

Our previous demonstration that F/B measured in tumor-stroma interface is prognostic of 10-year metastasis-free survival in a cohort of untreated breast cancer patients was performed in primary tumor excision sections [33]. That work suggests that F/B measured in tumor-stroma interface from primary tumor excisions may help predict metastasis and therefore improve treatment decisions in a clinical setting. Here, we have shown that F/B measured in the tumor-stroma interface of core needle biopsies from two different breast cancer subtypes is the same as F/B in the tumor-stroma interface of primary tumor excisions after NACT. The fact that this prognostic indicator was unchanged from (early) biopsy to (later) excision implies that F/B-based analysis of metastatic risk may be possible earlier in the patient’s timeline when a core needle biopsy is taken several months prior to NACT and tumor excision. This additional information, provided earlier, may prove useful in designing treatment plans. This study also found that NACT increased fiber alignment in TNBCs. This phenotype is associated with lymph node metastasis, we note that this stromal signature may also provide an earlier indicator of poor patient prognosis. Taken together, our results suggest that extracellular collagen parameters may add prognostic information earlier in a breast cancer patient’s treatment timeline.

## 5. Conclusions

Neoadjuvant chemotherapy (NACT) is often prescribed prior to surgical removal of aggressive breast tumors. NACT is known to have debilitating side effects and induces changes in prognostic indicators in the tumor microenvironment, which may encourage the development of metastases. In this study, we used second-harmonic generation (SHG) imaging to assess NACT-induced changes in two ECM-based prognostic indicators: F/B and fiber organization. We compared the SHG forward-to-backward-scattered ratio, sensitive to collagen fiber internal structure (FIS), in pre-treatment core needle biopsies and post-NACT tumor excisions. We found that collagen FIS is altered in the tumor bulk but not in the adjacent tumor-stroma interface of both HER2+ and TNBCs. Overall organization in collagen fiber orientation was not significantly changed by NACT in HER2+ tumors but was in TNBCs. Hence, the effect of NACT on these two ECM-based prognostic indicators is complex, with one parameter (organization) being altered in one tumor type (TNBC) but not the other (HER2+), while the other prognostic parameter (F/B from the tumor-stromal interface) was not altered in either subtype. This suggests that the effects of individual NACT drugs on these tumor extracellular matrix properties and any downstream effects on metastatic outcome are worthy of further study. Furthermore, as our previous studies reveal that F/B from the tumor-stroma interface of breast tumor excisions is prognostic of metastasis-free survival, and tumor-stroma interface F/B in biopsies is not different from tumor-stroma interface F/B in excisions, therefore, SHG F/B may be useful for assessing long-term patient outcome using diagnostic biopsies, prior to any surgery or chemotherapy treatments.

## Data Availability

The data acquired and analyzed in this study are available from the authors upon reasonable request.

## Supplementary Materials

The following supporting information can be downloaded at: https://www.mdpi.com/article/10.3390/cancers14040857/s1, Table S1: Clinical parameters of HER2+ and TNBC patient cohorts. Patient data and primary tumor characteristics (HR expression, nuclear grade) were measured and recorded by board-certified pathologists prior to NACT administration and tumor resection.
