# Supplementary material for: Second-Harmonic Generation Imaging Reveals Changes in Breast Tumor Collagen Induced by Neoadjuvant Chemotherapy
Source: Cancers (Basel). 2022 Feb 9;14(4):857. doi: 10.3390/cancers14040857 (PMC8869853; doi:10.3390/cancers14040857)
Supplement: Supplementary file 1 [file cancers-14-00857-s001.zip › cancers-1586932-supplementary.pdf]

Supplementary materials: Second–Harmonic Generation Imaging Reveals Changes in Breast Tumor Collagen Induced by Neoadjuvant Chemotherapy

Danielle E. Desa, Wencheng Wu, Robert M. Brown, Edward B. Brown IV, Robert L. Hill, Bradley M. Turner and Edward B. Brown III

Table S1. Clinical parameters of (a) HER2+ and (b) TNBC patient cohorts. Patient data and primary tumor characteristics (HR expression, nuclear grade) were measured and recorded by board–certified pathologists prior to NACT administration and tumor resection.

(a)

HER2+			
**Characteristic**		**No. Patients**	**%**
**Age (years)**	<40	3	13.64
	40–50	6	27.27
	50–60	6	27.27
	60–70	5	22.73
	70–80	0	0.00
	>80	1	4.55
	Unknown	1	4.55
**Nuclear grade**	1	2	9.09
	2	6	27.27
	3	14	63.64
**ER expression**	+	5	22.73
	−	16	72.73
**PR expression**	+	6	27.27
	−	15	68.18
**No HR data**		1	4.55

(b)

Triple Negative			
**Characteristic**		**No. Patients**	**%**
**Age (years)**	<40	4	18.18
	40–50	6	27.27
	50–60	6	27.27
	60–70	2	9.09
	70–80	3	13.64
	>80	1	4.55
**Nuclear grade**	2	1	4.55
	3	21	95.45
**CK5 expression**	+	6	27.27
	−	14	63.64
	Inconclusive	2	9.09
**EGFR expression**	+	7	31.82
	−	14	63.64
	Inconclusive	1	4.55
**Basal type**	Basal	7	31.82
	Nonbasal	15	68.18
